# Preoperative Bacteriuria and Antimicrobial Resistance in Men Undergoing Urethral Stricture Surgery

**DOI:** 10.3390/antibiotics15050472

**Published:** 2026-05-06

**Authors:** Mikołaj Frankiewicz, Łukasz Białek, Adam Kaftan, Marta Rydzińska, Jakub Dobruch, Michał Skrzypczyk, Marcin Matuszewski

**Affiliations:** 1 Department of Urology, Medical University of Gdansk, 80-210 Gdansk, Poland; mfrankiewicz@gumed.edu.pl (M.F.); adam.kaftan@gumed.edu.pl (A.K.); matmar@gumed.edu.pl (M.M.); 2 Department of Urology, Centre for Postgraduate Medical Education, 01-813 Warsaw, Poland; martaaga.drazkiewicz@gmail.com (M.R.); jakub.dobruch@cmkp.edu.pl (J.D.); mskrzypczyk@cmkp.edu.pl (M.S.)

**Keywords:** urethral stricture, urethroplasty, bacteriuria, antimicrobial prophylaxis, antimicrobial resistance, suprapubic catheter

## Abstract

**Background/Objectives**: Perioperative antibiotics are standard in urethral stricture surgery, but timely uncontaminated preoperative urine cultures are not always available, and empiric regimens are often selected before current susceptibility data are known. We aimed to characterize preoperative bacteriuria and antimicrobial resistance in men undergoing urethral stricture surgery and to assess associations with recorded clinical variables. **Methods**: We retrospectively analyzed 304 men undergoing urethral stricture surgery at two referral centers, representing the subset of the surgical population for whom a complete preoperative microbiology report, including antimicrobial susceptibility data when culture-positive organisms were recovered, was retrievable from the institutional microbiology laboratory information systems. Clinical contributors were assigned by structured chart review. The primary microbiologic endpoint was preoperative bacteriuria, defined by final local laboratory interpretation of the urine culture. Urine culture was requested at admission and repeated within 24 h before surgery if missing or non-diagnostic. MDR was defined as resistance to three or more clinically relevant antimicrobial classes, excluding expected intrinsic resistance patterns. **Results**: Among patients with retrievable complete preoperative microbiology data, urine cultures were positive in 164/304 patients (53.9%). The most frequent recorded contributors were iatrogenic exposure (119; 39.1%), prior endoscopic treatment (71; 23.4%), and trauma (64; 21.1%); 108/304 patients (35.5%) had more than one contributor. Among culture-positive patients, MDR occurred in 18/164 (11.0%). On exploratory multivariable analysis, suprapubic catheter status was independently associated with culture positivity (adjusted OR 4.41, 95% CI 2.47–7.87; *p* < 0.001), whereas PFUI was not significant after adjustment. **Conclusions**: In this analytic cohort, preoperative bacteriuria was common, and suprapubic catheter status was the strongest independent correlate of urine culture positivity. The observed MDR burden supports recent preoperative urine culture acquisition and stewardship-based perioperative antibiotic selection.

## 1. Introduction

Male urethral stricture disease can substantially impair urinary function and quality of life, and urethroplasty is the definitive reconstructive treatment for many patients [1]. Despite generally favorable functional outcomes, urethral reconstruction requires mucosal incision, tissue dissection, urinary diversion, and postoperative catheterization—features that create a clinically relevant connection between the operative field and genitourinary microbiology. Accordingly, perioperative antimicrobial strategies are embedded in contemporary guidance. The American Urological Association (AUA) recommends treating active urinary tract infection before intervention and obtaining a preoperative urine culture to guide antibiotic choice; it further advises limiting prophylaxis to a single dose or discontinuation within 24 h, with extension reserved for specific indications such as active infection or an indwelling suprapubic catheter. Similarly, the European Association of Urology (EAU) guidelines recommend preoperative urine culture and management of bacteriuria prior to procedures that breach the urinary tract mucosa, consistent with antimicrobial stewardship principles [2,3,4,5].

However, obtaining a timely, uncontaminated preoperative urine culture in urethral stricture disease is often difficult. This challenge is amplified by the referral-based nature of care: many patients are evaluated and treated at tertiary reconstructive centers and must travel for surgery, which can limit repeat sampling or prompt recollection in case of contamination. In addition, preoperative cultures in this setting are usually derived from voided or catheter-obtained urine and therefore most directly reflect bladder bacteriology rather than the urethral scar itself. As a result, perioperative prophylaxis is frequently selected according to institutional protocols or recent microbiology rather than same-day culture-directed susceptibility, particularly when cultures are negative, contaminated, delayed, or unavailable in time to guide incision-time decision-making [6]. Given the global rise in antimicrobial resistance, reducing unnecessary exposure while avoiding inadequate coverage remains a practical challenge. This issue is clinically relevant because available urethroplasty literature suggests that preoperative bacteriuria may identify patients at increased risk of short-term postoperative morbidity, while the relationship between urinary culture positivity and long-term reconstructive success remains uncertain [7,8,9]. Male urethral stricture disease is etiologically heterogeneous, and clinically relevant contributors frequently coexist in the same patient rather than presenting as discrete mutually exclusive categories [10,11].

The aim of the present study was to characterize the prevalence of preoperative bacteriuria and antimicrobial resistance in men undergoing urethral stricture surgery and to assess whether suprapubic catheter status and other recorded clinical variables, including stricture etiology, were associated with preoperative urine culture positivity.

## 2. Results

### 2.1. Patient Cohort and Clinical Context

The analytic cohort comprised 304 men undergoing urethral stricture surgery at two referral centers with expertise in reconstructive urology. At both centers, preoperative urine culture is requested as standard preoperative practice for all patients undergoing urethral stricture surgery. The present analysis was restricted to the subset of the surgical population for whom a complete preoperative microbiology report, including antimicrobial susceptibility data when culture-positive organisms were recovered, was retrievable from the institutional microbiology laboratory information systems, spanning 2017–2023. Median age was 57 years (IQR 40–68) and median stricture length was 15 mm (IQR 10–25; range 2–120 mm). Recorded clinical contributors included iatrogenic exposure (119; 39.1%), prior endoscopic treatment (71; 23.4%), trauma not classified as PFUI (64; 21.1%), idiopathic disease (62; 20.4%), hypospadias-associated disease (48; 15.8%), and PFUI (37; 12.2%). Because multiple plausible contributors could be present in the same patient, these variables were not treated as mutually exclusive etiologic groups; 108/304 patients (35.5%) had more than one recorded contributor. Accordingly, they were analyzed as overlapping clinical context flags.

### 2.2. Preoperative Urine Culture, Pathogens, and Polymicrobial Growth

**Figure 1:**
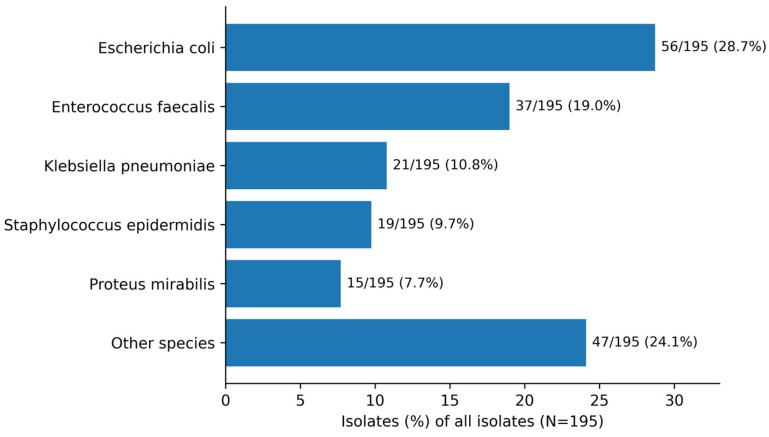
Distribution of organisms recovered from preoperative urine cultures among culture-positive patients (N = 195 isolates). Values are shown as n/195 (%).

**Figure 2:**
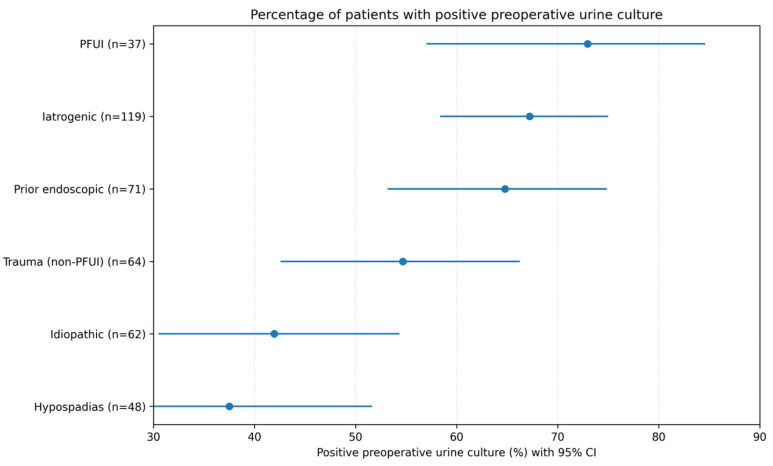
Proportion of patients with positive preoperative urine culture according to recorded clinical stricture context flags (Wilson 95% CI). Categories were not mutually exclusive; therefore, these comparisons are descriptive.

Among patients with retrievable complete preoperative microbiology data, 164/304 (53.9%) had a positive preoperative urine culture, whereas 140/304 (46.1%) had a negative culture. Polymicrobial growth was identified in 31/164 culture-positive patients (18.9%). Among the 31 polymicrobial cultures, two Gram-negative organisms were co-isolated in 19/31 (61.3%), with *Escherichia coli* and *Pseudomonas aeruginosa* as the most frequent specific combination (*n* = 3); Gram-negative + *Enterococcus* combinations occurred in 5/31 (16.1%), including *E. coli* + *E. faecalis* in 2 cases. Skin- or contamination-pattern flora (coagulase-negative staphylococci or *Staphylococcus epidermidis*) co-occurring with Gram-negatives were identified in 4/31 cultures, consistent with catheter-associated colonization. Across 195 isolates, the most frequently identified organisms were *Escherichia coli* (28.7%), *Enterococcus faecalis* (19.0%), *Klebsiella pneumoniae* (10.8%), and *Staphylococcus epidermidis* (9.7%) (Figure 1). The proportion of patients with a positive preoperative urine culture differed across recorded clinical context flags (Figure 2). On univariable analysis, PFUI was associated with higher odds of a positive culture compared with non-PFUI patients (OR 2.56, 95% CI 1.19–5.50; *p* = 0.02). Suprapubic catheter status was strongly associated with culture positivity (OR 4.83, 95% CI 2.96–7.88; *p* < 0.001). In an exploratory multivariable logistic model including recorded clinical context flags, age, stricture length, and suprapubic catheter status, suprapubic catheter status remained independently associated with culture positivity (adjusted OR 4.41, 95% CI 2.47–7.87; *p* < 0.001), whereas the PFUI association attenuated and was no longer statistically significant (adjusted OR 1.51, 95% CI 0.50–4.58; *p* = 0.46). These findings suggest that preoperative bacteriuria in this cohort was more closely linked to catheter exposure and healthcare-associated complexity than to PFUI itself as an isolated subtype.

### 2.3. Antimicrobial Resistance

Among patients with bacteriuria on preoperative urine culture, cephalosporin resistance was most frequent (20.5%), followed by trimethoprim–sulfamethoxazole (14.4%) and fluoroquinolones (13.3%), whereas resistance to aminoglycosides (1.6%) and carbapenems (0.0%) was uncommon (Table 1).

MDR was present in 18/164 culture-positive patients (11.0%). When summarized across recorded clinical context flags, MDR appeared more frequent in trauma-related groups, including PFUI and trauma without PFUI (Table 2). Because clinical context flags were not mutually exclusive, these summaries should be interpreted as descriptive rather than as inferential between-group comparisons. Intrinsic or non-informative resistance patterns characteristic of *Enterococcus* spp., including cephalosporin resistance, intrinsic low-level aminoglycoside resistance, and trimethoprim–sulfamethoxazole non-susceptibility, were not counted toward MDR classification, in line with the Magiorakos framework [12]. No *E. faecalis* isolate met MDR criteria after accounting for intrinsic resistance patterns. Specific resistance phenotypes of stewardship relevance were also examined where laboratory data permitted. In the final microbiology reports, no ESBL-producing *E. coli* isolate was identified, whereas two *K. pneumoniae* isolates were reported as ESBL-producers by the local microbiology laboratory. Resistance to third-generation cephalosporins was also reviewed descriptively: resistance to ceftriaxone or ceftazidime was present in 5/45 (11.1%) *E. coli* isolates and 8/19 (42.1%) *K. pneumoniae* isolates with interpretable third-generation cephalosporin susceptibility. No vancomycin-resistant *Enterococcus* was identified among isolates with interpretable vancomycin susceptibility.

## 3. Discussion

Perioperative antimicrobial prophylaxis in urethral stricture surgery lies at the intersection of reconstructive complexity and infectious risk. Both the AUA and EAU recommend preoperative urine culture acquisition and treatment of active bacteriuria before procedures that breach the urinary tract mucosa, reflecting the well-established principle that culture-directed prophylaxis reduces perioperative infectious morbidity [2,3,4,5,13,14]. In practice, however, obtaining a timely and uncontaminated preoperative culture in this population is often difficult: patients are commonly referred to tertiary reconstructive centers, may harbor chronic indwelling suprapubic catheters, and frequently present with a microbiologic history shaped by repeated instrumentation rather than community-acquired exposure. Empiric perioperative antibiotic selection is therefore common, making characterization of the preoperative microbiologic landscape—including bacteriuria prevalence, pathogen distribution, and resistance patterns—directly relevant to perioperative planning and antimicrobial stewardship in reconstructive urology.

In the present cohort of 304 men undergoing urethral stricture surgery, preoperative bacteriuria was identified in more than half of patients (53.9%). The dominant signal in the adjusted analysis was not a specific etiologic subtype but suprapubic catheter exposure, which remained independently associated with culture positivity, whereas the PFUI association attenuated after adjustment. These findings suggest that preoperative bacteriuria in this setting is linked predominantly to catheter exposure and healthcare-associated complexity rather than to any single etiologic label. This interpretation is clinically plausible: patients with chronic urinary diversion or repeated instrumentation are more likely to develop persistent bacteriuria, polymicrobial colonization, and antimicrobial resistance because of repeated healthcare exposure and biofilm formation [15]. Accordingly, clinical attention should be directed toward catheter exposure as the primary determinant of microbiologic risk. A recent retrospective study of patients with urethral stricture and indwelling suprapubic catheters documented that catheter-associated bacteriuria was common and MDR organisms were augmented among patients with longer catheter duration and additional risk factors. Our findings are therefore consistent with the broader catheter-associated urinary tract infection literature and contemporary urologic infection guidance [16,17]. The 11.0% MDR burden observed in our culture-positive cohort is broadly comparable to recent reports from catheter-associated and reconstructive urology populations, in which MDR rates of approximately 10–20% have been described among patients with prolonged catheter exposure, repeated instrumentation, or healthcare-associated urinary colonization [16,17]. Our results sit toward the lower end of this range, which likely reflects the heterogeneous case mix of urethroplasty patients, in whom only a subset carry long-term suprapubic catheters at the time of surgery.

This association is also biologically plausible because indwelling catheters provide a substrate for biofilm formation, and adherent organisms may be incompletely represented by routine urine culture. In practical terms, catheter biofilm may act as a persistent microbial reservoir that helps explain ongoing bacteriuria, polymicrobial growth, and occasional discordance between culture results and the true perioperative microbial environment [18,19]. Emerging stricture-specific microbiome data further suggest that uropathogenic dysbiosis and biofilm-forming organisms may contribute to a pro-inflammatory local environment, although direct evidence that biofilm independently worsens urethroplasty healing or recurrence remains limited [20].

Guidelines emphasize short-course prophylaxis (single dose or discontinuation within 24 h) and selection informed by cultures and local resistance patterns, with extension reserved for specific indications such as active infection or selected catheter scenarios [2,3,4]. Nevertheless, practice variation remains substantial. A survey of antimicrobial practice patterns for urethroplasty identified heterogeneous approaches to both perioperative and postoperative antibiotics, highlighting an opportunity for improved stewardship in reconstructive urology [21]. More recent outcome data align with this stewardship strategy: postoperative prophylactic antibiotic use after urethral reconstruction has been associated with higher rates of MDR colonization or infection without clear improvement in clinical outcomes [22].

Our findings support a pragmatic stewardship-oriented framework. Preoperative urine culture remains the preferred basis for perioperative antimicrobial planning whenever feasible. When current culture results are unavailable, delayed, or non-diagnostic, the most clinically relevant warning signs in this dataset appear to be suprapubic catheterization and broader healthcare-associated complexity rather than any single etiologic label alone. In such patients, obtaining a recent preoperative culture as close to surgery as possible and aligning empiric prophylaxis with local resistance patterns for catheter-associated bacteriuria may be more rational than relying on standard empiric perioperative prophylaxis assumptions [2,3,4,13,23,24].

An important next step would be prospective evaluation of whether preoperative suprapubic catheter exchange followed by culture collection from the newly placed catheter improves the clinical relevance of preoperative microbiology. This approach is biologically plausible because long-standing catheters rapidly develop biofilm and polymicrobial colonization, which may distort culture results and reduce concordance with true bladder bacteriology [11]. A prospective study comparing standardized catheter exchange and fresh-catheter sampling with usual-care sampling from the existing catheter would therefore be justified.

This study has several limitations. First, the analytic cohort was restricted to patients with a retrievable complete preoperative microbiology report from the institutional microbiology laboratory information systems. The reported bacteriuria prevalence of 53.9% should therefore be interpreted as applying to the analytic cohort rather than as the prevalence among all men undergoing urethral stricture surgery at the participating centers. Second, non-diagnostic urine collections—most commonly due to contamination—do occur in routine practice; sample-level contamination flags were performed at the local microbiology laboratory level and only final reported results were abstracted into the study database, so the precise number of contaminated samples and patients undergoing repeat sampling was not enumerated retrospectively. Contamination is primarily a sample-level technical issue; however, because sample-level contamination flags were not retained in the analytic dataset, we cannot exclude heterogeneity related to collection route, catheter status, or local workflow. Third, symptom status at the time of urine sampling was not consistently documented; therefore, the primary microbiologic endpoint should be interpreted as bacteriuria rather than symptomatic urinary tract infection. Fourth, the source of preoperative urine collection was not standardized across the study period, which may have introduced heterogeneity in culture findings; however, suprapubic catheter samples were collected only when voided urine could not be obtained. Finally, the microbiology dataset was based on standard clinical urine cultures and did not capture the full complexity of stricture-associated biofilm or urethral scar microbiology.

## 4. Materials and Methods

### 4.1. Study Design and Patient Cohort

We performed a retrospective cohort study of adult men undergoing urethral stricture surgery (urethroplasty/open urethral reconstruction) at two participating referral centers. At both centers, preoperative urine culture is requested as standard preoperative practice for all patients undergoing urethral stricture surgery, irrespective of catheter status, symptoms, or prior infectious history; when an initial sample was unavailable or reported as non-diagnostic, a repeat culture was obtained within 24 h before surgery. For the present analysis, the analytic cohort comprised 304 patients for whom a complete preoperative microbiology report was retrievable from the institutional microbiology laboratory information systems, including antimicrobial susceptibility data when culture-positive organisms were recovered, spanning 2017–2023. Patients without a retrievable complete preoperative microbiology report were not included. Because preoperative culture acquisition is requested as standard practice at both centers, inclusion in the present analysis reflects retrievability of the complete preoperative microbiology report rather than a clinical decision to obtain or omit culture testing. To ensure consistent microbiological methodology across the cohort, only patients whose preoperative urine cultures were processed and reported by the on-site clinical microbiology laboratory of one of the two participating centers were included; samples processed at external laboratories were not retained for analysis.

### 4.2. Clinical Context Variables

Recorded clinical contributors to stricture disease were assigned by structured review of the medical record, focusing on prior urethral instrumentation and procedures, history of endoscopic stricture treatment, traumatic injury, pelvic fracture urethral injury (PFUI), inflammatory conditions (including urethritis and lichen sclerosus/balanitis xerotica obliterans), hypospadias-associated disease, and idiopathic presentation when no plausible cause could be identified. Because more than one clinically relevant contributor could be present in the same patient, these variables were not treated as mutually exclusive etiologic groups. Instead, they were analyzed as overlapping descriptive clinical context flags.

### 4.3. Urine Culture Workflow, Endpoint, and Microbiology

Patients were asked to provide a urine sample for culture at hospital admission. In routine practice, samples were obtained from voided urine in patients able to void and from catheter-obtained urine in those with suprapubic drainage. When an initial sample was unavailable or reported as non-diagnostic, an additional urine culture was collected within 24 h before surgery. For the present analysis, the final preoperative culture was defined as the most recent valid, interpretable urine culture result available before surgery. The database captured the final reported preoperative culture result rather than the complete sample-level culture sequence; therefore, repeat-culture frequency and contamination rates could not be analyzed reliably. For the present study, the primary microbiologic endpoint was preoperative bacteriuria, defined as a urine culture reported as positive by the local microbiology laboratory. Symptom status at the time of sampling was not consistently documented; accordingly, this endpoint should be interpreted as bacteriuria rather than symptomatic urinary tract infection. Perioperative antibiotics at the time of incision were selected empirically when culture identification and susceptibility results were unavailable or when the submitted sample was non-diagnostic; in routine practice, these results were typically available 48–72 h after sample collection. Culture findings were abstracted as reported by the local microbiology laboratory. For descriptive microbiology, organism distribution was summarized at the isolate level, so polymicrobial cultures contributed more than one isolate. Antimicrobial susceptibility was interpreted using EUCAST clinical breakpoints; isolates reported as susceptible or susceptible with increased exposure were considered active against the recovered organism, whereas resistant isolates were considered inactive [14]. Both on-site clinical microbiology laboratories operated under EUCAST breakpoints throughout the study period and used routine identification and antimicrobial susceptibility testing methods consistent with national clinical microbiology practice, providing comparable analytic quality across the cohort.

### 4.4. Perioperative Antibiotics and Multidrug Resistance

Perioperative antibiotic regimens administered at the time of surgery were obtained from anesthesia and operative documentation and grouped into major antimicrobial classes. Multidrug resistance (MDR) was defined as non-susceptibility to at least one agent in three or more clinically relevant antimicrobial categories, consistent with international consensus definitions [12]. Expected intrinsic resistance patterns were not counted toward MDR classification. For *Enterococcus faecalis*, intrinsic or non-informative resistance patterns, including cephalosporin resistance, low-level aminoglycoside resistance, and trimethoprim–sulfamethoxazole resistance, were excluded from MDR category counts.

### 4.5. Statistical Analysis

Analyses were primarily descriptive. Continuous variables are summarized as median with interquartile range (IQR), and categorical variables as counts and percentages. Associations with preoperative culture positivity were assessed using Fisher’s exact tests and reported as odds ratios with 95% confidence intervals. An exploratory multivariable logistic regression model was fit to evaluate associations with culture positivity, including age, stricture length, recorded clinical context flags, and suprapubic catheter status. Two-sided tests were used with a significance threshold of 0.05. Analyses were performed in Stata/BE 18.0 (StataCorp LLC, College Station, TX, USA).

## 5. Conclusions

In this analytic cohort of men undergoing urethral stricture surgery with retrievable preoperative microbiology data, preoperative bacteriuria was frequent, with suprapubic catheter status representing the strongest independent correlate of culture positivity. The accompanying multidrug-resistant burden supports routine acquisition of a recent preoperative urine culture and culture-guided, stewardship-based perioperative antibiotic selection rather than standardized empiric prophylaxis.

## Figures and Tables

**Table 1:** Antimicrobial resistance by class and multidrug resistance among culture-positive patients.

Antimicrobial Class	Resistant Isolates n/N (%)
Cephalosporins	23/112 (20.5)
Trimethoprim–sulfamethoxazole	18/125 (14.4)
Fluoroquinolones	14/105 (13.3)
MDR (≥3 classes)	18/164 (11.0)
Aminoglycosides	2/122 (1.6)
Carbapenems	0/117 (0.0)

**Table 2:** Descriptive summary of preoperative urine culture positivity and multidrug resistance according to recorded clinical stricture context flags. Categories were not mutually exclusive; therefore, totals exceed 304 and comparisons are descriptive.

Clinical Context Flag	Culture + n/N (%)	MDR n/N (%)
PFUI	27/37 (73.0)	6/27 (22.2)
Iatrogenic	80/119 (67.2)	9/80 (11.3)
Prior endoscopic	46/71 (64.8)	6/46 (13.0)
Trauma (non-PFUI)	35/64 (54.7)	8/35 (22.9)
Hypospadias-associated	18/48 (37.5)	1/18 (5.6)
Idiopathic	26/62 (41.9)	2/26 (7.7)

## Data Availability

The data presented in this study are available on reasonable request from the corresponding author. The data are not publicly available due to privacy and ethical restrictions.
